# Optimizing Exclusion Criteria for Clinical Trials of Persistent Lyme Disease Using Real-World Data

**DOI:** 10.3390/healthcare13010020

**Published:** 2024-12-25

**Authors:** Lorraine Johnson, Mira Shapiro, Deanna Needell, Raphael B. Stricker

**Affiliations:** 1 LymeDisease.org, Los Angeles, CA 91040, USA; lorrainejohnson@outlook.com; 2 Analytic Designers LLC, Bethesda, MD 20817, USA; mira.shapiro@analyticdesigners.com; 3 Department of Mathematics, University of California, Los Angeles, CA 90025, USA; deanna@math.ucla.edu; 4 Union Square Medical Associates, 595 Buckingham Way, Suite 350, San Francisco, CA 94132, USA

**Keywords:** persistent Lyme disease, chronic Lyme disease, PTLDS, real-world data, eligibility criteria, enrollment, recruitment, generalizability, big data, sample yield

## Abstract

Background/Objectives: Although eligibility criteria for clinical trials significantly impact study outcomes, these criteria are often established without scientific justification, leading to delayed recruitment, small sample sizes, and limited study generalizability. Persistent Lyme disease (PLD) presents unique challenges due to symptom variability, inconsistent treatment responses, and the lack of reliable biomarkers, underscoring the need for scientifically justified eligibility criteria. Objective: This study examines the effects of commonly used enrollment criteria on sample yield in PLD clinical trials using real-world data (RWD) from the MyLymeData patient registry. The study also compares the effects of these criteria on enrollment for PLD versus acute Lyme disease (ALD) trials and evaluates the scientific rationale for each criterion. Methods: Data from 4183 Lyme disease patients enrolled in the MyLymeData registry were analyzed to assess the prevalence and cumulative impact of various criteria on sample yield. A comparative analysis of cohorts with PLD (n = 3589) versus ALD (n = 594) was conducted to identify differences in sample attrition. Results: In a large PLD cohort study, we found that current commonly used eligibility criteria would exclude approximately 90% of patients, significantly limiting study generalizability. Substantial differences in sample attrition between PLD and ALD cohorts highlight the need for tailored criteria. The strength of scientific justification varied widely among criteria. Conclusions: This study demonstrates the importance of using RWD to optimize eligibility criteria in PLD clinical trials. By providing insights into the balance between sample attrition and scientific justification, researchers can enhance trial feasibility, generalizability, and robustness. Our RWD sample demonstrates that researchers could substantially increase the sample yield from 10% to 64% by loosening restrictions on coinfections and misdiagnoses of chronic fatigue syndrome, fibromyalgia syndrome, and psychiatric conditions.

## 1. Introduction

Lyme disease affects nearly half a million new patients annually in the United States [1,2]. While many patients with acute Lyme disease (ALD) present with an erythema migrans (EM) rash and recover with timely treatment, up to 43% report persistent symptoms six months after treatment, a condition often termed persistent or chronic Lyme disease (PLD) [3]. Common clinical manifestations of PLD in adults include musculoskeletal pain, neurological impairment, and heart-related conditions [4]. An estimated two million Americans have PLD, with 72% reporting fair or poor health status, compared to 13% in the general population [5,6]. The disease is also linked with a higher risk of suicide [7].

PLD is a broad diagnostic term that encompasses patient populations treated clinically [8]. In contrast, the term post-treatment Lyme disease syndrome (PTLDS) is a research definition that is primarily used by academic researchers to reflect a much smaller subgroup of the clinical population that remains after applying exclusion criteria to a study. Hence, PTLDS samples represent a highly restrictive subset of patients with PLD [8,9]. While patients with PLD are thought to have unresolved sequelae of infection with *Borrelia burgdorferi*, the spirochetal agent of Lyme disease, reinfection is also possible and may be accompanied by a new EM rash [8].

Although clinical trials aim to improve patient outcomes, restrictive eligibility criteria often limit their generalizability. This issue, which is prevalent across medical research, contributes to recruitment challenges, underpowered trials, increased costs, and reduced external validity [10,11,12,13,14]. The gap between academic research and community clinical practice has resulted in a critical lack of clinical guidance for treatment [15]. These problems have led some researchers to declare a crisis of confidence in the clinical trial system [16]. A key issue lies in eligibility criteria that yield samples that are not representative of clinical populations [15,17,18]. Many studies adopt criteria without reassessing the strength of their scientific justification, a practice deemed “suboptimal”, “subjective”, and “unsystematic” [11,12,13,19]. A review of 283 studies found that numerous exclusion criteria were unjustified, underscoring the need for a more data-driven approach [19].

The use of small, unrepresentative trials is among the most significant challenges in PLD research. Only four NIH-funded randomized controlled trials (RCTs) for PLD treatments have been conducted [20,21,22]. These trials were conducted over two decades ago, excluded between 88 and 99% of patients who sought to enroll, and took between 2.5 and 4 years to recruit [23]. The U.S. Food and Drug Administration (FDA) has advocated for less restrictive entry criteria and further scientific justification for each criterion [24]. Real-world data (RWD) from big data sources, including patient registries, now allow researchers to assess the impact of eligibility criteria on study populations [13,18,25]. However, unlike other diseases, no studies have evaluated the external validity of RCT results in PLD using RWD.

To address this gap, we have analyzed data from 4183 patients in the MyLymeData registry, which includes comprehensive data often missing from other big data sources such as electronic health records (EHRs) or insurance claims data [23]. Additionally, we have used machine learning to predict symptoms associated with the misdiagnosis of chronic fatigue syndrome (CFS) or fibromyalgia syndrome (FMS) (collectively CFS/FMS), which may lead to inappropriate exclusions. Our findings aim to inform more inclusive and representative trial designs, thereby improving the quality and applicability of PLD research. By identifying trade-offs between internal and external validity in eligibility criteria, we seek to encourage a balanced approach in study design. This should lead to more robust, generalizable studies, providing clinicians with evidence-based guidance for effective PLD treatment.

## 2. Materials and Methods

### 2.1. Data Source

This study analyzed the responses of participants who completed Phase 2 of the MyLymeData patient registry project as of 21 August 2022. MyLymeData was launched in 2015. Participant responses are recorded in the registry, which includes symptoms, diagnostic testing, functional impairment, treatment response, and side effects. Survey questions were developed from a combination of government survey items derived from the Agency for Healthcare Research and Quality (AHRQ), National Health Interview Survey (NHIS), the CDC Behavioral Risk Factor Surveillance System (BRFSS), the Centers for Disease Control and Prevention (CDC) Healthy Days Measures, the peer-reviewed literature for Lyme disease, and a database of 51 other patient registries that were included in the Patient-Centered Outcomes Research Institute (PCORI) patient registry project. The survey was beta-tested with clinicians and patients and modified as appropriate.

Recruitment for participation in the survey was accomplished through blogs and social media, word of mouth, as well as professional and government conference presentations about the registry. All respondents participate in the registry voluntarily and their identities remain strictly confidential. Written informed consent for participation was obtained from all patients involved in the study. The MyLymeData patient registry research study (#LD12015) was initially approved by the Advarra Institutional Review Board, Columbia, MD, in 2015 (Pro00014923) and approval for the study has been renewed annually. In addition, the analysis of the survey data for the study was exempted from review in 2017 by the University of California Los Angeles Institutional Review Board (IRB#17-000933) because it did not meet the definition of direct human subject research.

Six peer-reviewed articles based on survey data have been published [6,9,23,26,27,28]. An overview of the survey items has been described in a previous publication [23].

### 2.2. Study Participants

**Figure 1:**
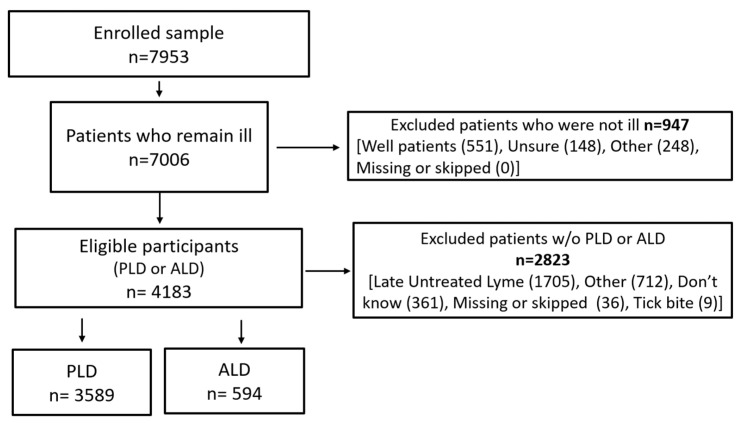
Inclusion and exclusion criteria for initial study sample. MyLymeData, Phase 2, 21 August 2022.

As illustrated in Figure 1 below, patients included in the sample (4183) for this study were US residents who reported being diagnosed with Lyme disease by a clinician and also reported their current status as ill (as opposed to being well). We then selected subgroups of patients based on stage of disease:

Patients included in the ALD subgroup reported a rash (irregular or bullseye) and/or Lyme disease symptoms regardless of whether they had received any treatment (n = 594). These patients had symptoms of Lyme disease for less than six months. Patients included in the PLD subgroup were further along in their disease progression, reporting that they had received antibiotic treatment and remained ill for 6 or more months following that treatment (n = 3589).

The demographics for the patients included in the study are detailed in Table 1. These descriptive statistics conform with other analyses using the MyLymeData patient registry.

### 2.3. Methodology Overview

The study was designed to evaluate the effect of eligibility criteria commonly used by academic researchers. In 2006, the IDSA proposed a research definition of PLD, modifications of which have been operationalized for various studies and used as eligibility criteria [3,29,30]. Generally, these criteria require evidence of prior Lyme disease, the presence of specific symptoms and functional impairment for at least six months after initial treatment, and the absence of certain comorbidities. Although the IDSA-proposed research definition of PLD contains over 40 eligibility criteria, only a small number of these are used in operational definitions. These include the following:

- Requiring that patients meet the CDC surveillance case definition, have a CDC Western blot positive lab test or physician-diagnosed EM, and report characteristic symptoms of Lyme disease of such severity that they result in functional impairment.
- Excluding patients with a prior diagnosis of most common psychiatric conditions, CFS or FMS, or a diagnosis with a tick-borne coinfection.

The MyLymeData registry survey items include commonly used eligibility questions that determine the effects on sample yield. Our analysis relies on patient-reported responses to questions in the MyLymeData patient registry (US residents, diagnosed by a physician, and reported their stage of illness as PLD or ALD). This analysis further takes into account the effect of misdiagnosis of CFS/FMS, patient-reported moderate to very severe symptoms, and functional impairment.

Although our survey questions are not identical to other commonly used research criteria, they are similar. For example, we assessed activity limitations based on the presence of one or more activity limitation days in a 30-day period (derived from a commonly used CDC Healthy Days measure) whereas some researchers have used 2 or more days in a 14-day period [31,32]. We assessed the impact of these eligibility criteria on sample attrition on an individual basis (prevalence) and cumulative basis (sample yield).

For a portion of the study, we used a separate modified sample (Attrition Sample) to analyze the impact of commonly used eligibility criteria on sample yield. The Attrition Sample included only patients who responded with an analyzable answer (eliminating missing or “don’t know” responses) to establish a uniform sample size for the sample yield analysis. The size of the Attrition Sample was 2786 (PLD 2396, ALD 400). (See Supplementary Materials).

These questions are as follows:

“Many patients with Lyme disease develop a bull’s eye or irregular rash. When I contracted Lyme disease”: included patients who responded that they did not have a rash or that they had a bullseye or irregular rash;

“My Lyme disease diagnosis is supported by positive laboratory testing (such as Western blot or Elisa)”: included patients who responded Yes or No;

“I would CURRENTLY describe the severity of my individual symptoms of Lyme disease as…”: included patients who indicated a response for at least one symptom;

“Before being diagnosed with Lyme disease, I was misdiagnosed with another condition”: included patients who responded Yes or No.

We also asked patients about coinfections and activity limitations:

“I have been diagnosed with a tick- borne coinfection” (yes/no).

“During the PAST 30 DAYS, I was kept from doing usual activities because of poor physical or mental health due to Lyme disease for about…” (0 to 30 days).

We evaluated the sample yield impact of the commonly used eligibility criteria on the PLD and ALD subgroups. This analysis was conducted in a stepwise fashion: (a) clinically diagnosed, (b) at least one moderate to very severe symptom (grouped none or mild vs. moderate to very severe), (c) the presence of either a rash or CDC-positive Western blot (Rash/WB+), and (d) patients who would inappropriately be excluded based on a previous misdiagnosis of CFS or FMS. We further assessed the impact of excluding patients diagnosed with a tick-borne coinfection and evaluated the impact of activity limitations.

To gain a better understanding of the high prevalence of misdiagnosis with CFS/FMS, we applied a semi-supervised machine learning (ML) method, non-negative matrix factorization, to determine which symptoms were most predictive of a CFS/FMS misdiagnosis in the PLD patient subgroup.

All statistical significance tests between ALD and PLD subgroup eligibility criteria were performed using the nonparametric Fisher’s Exact Test with alpha = 0.05. We chose the nonparametric Fisher’s Exact Test, which allows for assessment of the association between two categorical variables by calculating the exact probability of the observational data but does not require distribution assumptions [9]. All analyses were performed using Python 3.12 (Python Software Foundation, Beaverton, OR, USA) and JMP^®®^ 18.0, (JMP Statistical Discovery LLC, Cary, NC, USA).

## 3. Results

### 3.1. Prevalence of Commonly Used Eligibility Criteria in the MyLymeData Sample

Table 2 below details the prevalence of commonly used eligibility factors as well as quality-of-life indicators compared across our two subgroups.

As shown in Table 2 above, PLD patients were slightly more likely to report at least one moderate to very severe symptom than ALD patients (98% vs. 93%). PLD patients were less likely than ALD patients to report a rash or WB+ test result (67% vs. 73%). PLD patients were more likely than ALD patients to be misdiagnosed (74% vs. 43%), and more specifically, to be misdiagnosed with CFS/FMS (40% vs. 6%) or psychiatric conditions (38% vs. 11%) and to be diagnosed with at least one coinfection (76% vs. 34%).

We compared quality-of-life indicators for the PLD and ALD subgroups. PLD patients reported a substantially worse quality of life on all indicators. PLD patients are more than two times more likely than ALD patients (74% vs. 36%) to report their health status as fair or poor. More PLD patients report their work status as disabled as compared to ALD patients (28% vs. 6%). As indicated in Table 2, the measurable differences in all prevalence factors between PLD and ALD were statistically significant using Fisher’s Exact Test.

Our ML analysis of the PLD subgroup identified the most strongly correlated predictors of a CFS/FMS misdiagnosis to be the presence of fatigue, muscle aches, and sleep impairment. These symptoms are highly prevalent in patients from our PLD subgroup: fatigue (87%), muscle aches (76%), and sleep impairment (70%). This could explain why misdiagnosis of CFS/FMS is so high in this population. Sixty-three percent of PLD patients with a prior CFS/FMS misdiagnosis also reported either having an EM rash or a WB+ test result (n = 1229 PLD patients with CFS/FMS misdiagnosis and an analyzable response to Rash/WB+ questions). (See Supplementary Materials.)

### 3.2. Effect of Commonly Used Eligibility Criteria on Sample Attrition

**Figure 2:**
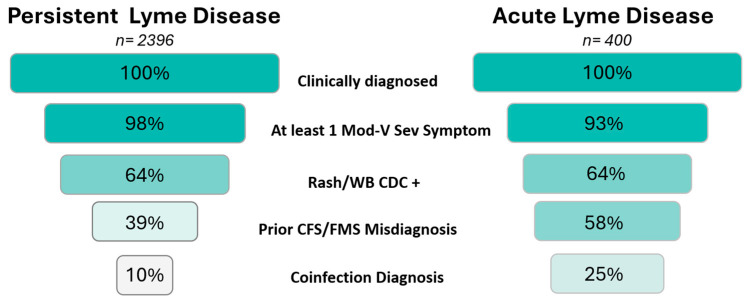
Sample yield reduction of PLD and ALD subgroups using Attrition Sample.

The effect of commonly used eligibility criteria on sample yield is shown below in Figure 2. Applying these eligibility criteria sequentially to the PLD subgroup, the sample yield is 10% compared to 25% for the ALD subgroup.

Eligibility criteria commonly exclude patients with a prior CFS/FMS diagnosis. However, a substantial portion of patients report that they were previously misdiagnosed with CFS/FMS. These patients should not be excluded. The figure reflects patients inappropriately excluded. Note that the cumulative effect of these criteria varies from prevalence percentages (see Table 2) because patients are excluded at different steps in the sequential application of eligibility criteria.

### 3.3. Analysis of Results

For clinical trials to produce results applicable to real-world practice, the trials must represent the full spectrum of patients seen by clinicians [10,11,15]. Exclusion criteria narrow the study population, potentially skewing results toward less complex cases with a more straightforward diagnostic journey. This process also reduces the sample size and yield, leading to under-recruitment, increased costs, and delayed trials [14,33].

PLD poses significant challenges for researchers due to its diverse symptoms and the lack of reliable biomarkers, underscoring the need for thoughtful eligibility criteria. Restrictive eligibility criteria often fail to reflect the heterogeneity of real-world patients, creating research gaps and leaving clinicians without adequate guidance for treatment [34].

Our findings, illustrated in Figure 2, using the Attrition Sample, demonstrate that commonly used eligibility criteria exclude 9 out of 10 patients from PLD clinical trials. Requiring a clinical diagnosis, a rash or CDC-positive Western blot, and at least one moderate to very severe symptom reduced the sample yield to 64%. Exclusion of patients misdiagnosed with CFS/FMS dropped the yield to 39%, and excluding those with coinfections reduced the yield to 10%. This unacceptably low sample yield is consistent with other PLD studies [3,20,21,22,35], while studies in other diseases typically have higher sample yields, averaging 65% [10].

Comparing the impact of these criteria on the PLD and ALD subgroups in the Attrition Sample, we found little difference in attrition when applying the first three criteria. However, excluding patients misdiagnosed with CFS/FMS had a more pronounced effect on PLD patients, as 40% of PLD patients had been misdiagnosed, compared to 8% of ALD patients. The same is true in psychiatric conditions, where 74% of PLD compared with 43% of ALD patients reported being previously misdiagnosed. A similar pattern was observed with coinfections, which were also more prevalent in PLD patients (PLD 76% vs. ALD 34%).

Clinicians often criticize the limited real-world applicability of RCT results when trials use overly restrictive criteria [15]. To optimize PLD research, eligibility criteria must be scientifically justified and designed to enhance both relevance and external validity.

## 4. Discussion

Many studies adopt eligibility criteria from previous research without thoroughly evaluating the rationale [11]. However, each individual exclusion decreases the sample yield and generalizability, as demonstrated in a PLD EHR study where adding an antibiotic prescription criterion reduced the sample size by 85% [36]. Carefully considering the impact of each eligibility criterion during study design is essential. Criteria should only be used when scientifically essential to achieving the study objectives [13,33].

Although Bechtold proposed some rationale for excluding specific comorbidities, no comprehensive assessment of the scientific validity of commonly used eligibility criteria has been conducted [29]. Below, we evaluate the scientific justification for key eligibility criteria and their effect on sample attrition.

### 4.1. Clinical Diagnosis and Symptoms

While requiring a clinical diagnosis and symptoms consistent with Lyme disease is crucial for participant selection in trials, some commonly used eligibility criteria go beyond this by requiring that patients meet the CDC’s surveillance case definition prior to treatment, followed by additional entry criteria. However, as noted by Dr. Mead of the CDC, the surveillance definition was designed for epidemiological purposes and is too restrictive for clinical diagnosis [23]. In the four NIH-funded PLD trials, the average sample yield was just 3.8%, with the primary cause of this high attrition being prior documentation of meeting CDC surveillance requirements [23,37].

The CDC itself acknowledges the limitations of its surveillance system and applies a multiplier when estimating the annual incidence of Lyme disease [38]. Many patients do not meet the stringent CDC surveillance requirements [39]. If we had initially required our sample to meet the CDC surveillance criteria, our PLD sample yield likely would have dropped by 50–90%, leaving only 1–5% of participants eligible. Taken together, this analysis provides strong evidence that the CDC surveillance definition requirements are excessively restrictive when used as eligibility criteria and introduce unnecessary barriers to recruitment that significantly reduce the potential pool of eligible participants, leading to a dramatic drop in sample yield, without scientific justification. This does not diminish the importance of maintaining the Rash/WB+ eligibility criteria, as discussed below.

Researchers should also consider clinical diagnosis and other clinically relevant diagnostic factors, such as characteristic signs and symptoms. Symptoms associated with Lyme disease are common in the general population, but their severity tends to be higher in Lyme patients [29]. To reduce overlap with other conditions, many studies require at least one moderate to very severe symptom [28,39,40]. In our study, this requirement had a negligible impact on sample attrition.

### 4.2. Rash/WB+

Requiring either an EM rash or a CDC+ Western blot is a common eligibility criterion; however, this requirement reduced the PLD sample by 33% because many Lyme patients do not develop a rash or have a CDC+ Western blot result [35,41]. While there is compelling rationale for having some criteria here as confirmation of the disease, it could be broadened to include other diagnostic tests and to consider patient-reported rashes as one of the self-reported items on their screening form.

### 4.3. Prior Misdiagnosis of CFS/FMS or Psychiatric Conditions

Some common eligibility criteria exclude patients with a history of CFS/FMS or psychiatric conditions. However, initial misdiagnosis is quite high in Lyme disease and disproportionately affects PLD patients. In our sample, 40% of PLD patients had a prior misdiagnosis of CFS/FMS and 38% had a psychiatric diagnosis. Misdiagnosis is a medical error, not a comorbidity, and should not be a basis for exclusion. Researchers can easily avoid sample attrition reflecting misdiagnosis in their eligibility screening questions by asking patients if they were previously misdiagnosed.

### 4.4. Coinfections

Excluding patients with coinfections further reduces the representativeness of PLD trials, as coinfections are more common in this population (76% of PLD patients versus 34% of ALD patients). Instead of excluding these patients, including them as a separate subgroup would yield more clinically meaningful data and improve understanding of the role coinfections play in PLD.

### 4.5. Functional Impairment

The use of functional impairment as an eligibility criterion in PLD clinical trials lacks strong justification and was not used in any conducted prior to 2006 [3,29]. Nor has any rationale for its use been presented in the literature [29]. Using this criterion may reduce sample sizes by up to 30% [29].

This criterion also may introduce potential bias in patient selection because some patients may adopt coping mechanisms, such as reducing work hours, changing the nature of their work, or adopting a position with flexible work hours. These compensatory strategies can mask the true level of impairment and become the “new normal” for patients that may not be accurately reflected in surveys with short look-back periods [6,32].

Moreover, functional impairment is not used by community clinicians for PLD diagnosis, further disconnecting research from real-world applicability. It also correlates with symptom severity [29]. This suggests that it offers little additional insight beyond that already provided by the upper range of symptom scales. Including only the most severe cases limits the spectrum of patients needed for robust analysis, making this criterion problematic.

### 4.6. Small Trials

Overly restrictive eligibility criteria in clinical trials can lead to small sample sizes, compromising the ability to detect meaningful treatment effects, particularly in conditions like PLD, where patient heterogeneity necessitates subgroup analysis generally and where treatments may benefit some patients significantly while not helping others [23,42]. This is an essential consideration when using patient-reported outcomes (PROs), as is common in PLD due to the lack of objective biomarkers [13,18].

In the context of PROs, clinical relevance is determined by the concept of minimal clinically important difference (MCID), which represents the smallest change in treatment outcome that a patient would consider important and that could influence clinical management [43]. During study design, the magnitude of change required to determine treatment success must be selected such that it can detect the smallest clinically relevant change in treatment outcome. Two of the four PLD treatment trials have been criticized for not using MCID and for requiring excessively large treatment effects to demonstrate success with small sample sizes [44]. Inadequate sample sizes not only affect individual studies but can also lead to apparent contradictions in the literature due to sampling variability [45]. Such contradictions have been noted in PLD research [44,46].

Additionally, restrictive criteria increase recruitment time, cost, and success—25% of RCTs are discontinued because of poor recruitment [47]. Investigator-initiated RCTs with smaller sample sizes are the most likely to be discontinued as a result of poor patient recruitment [47]. Economic constraints pose challenges for researchers, but carefully selecting eligibility criteria in the research design will ultimately yield larger sample sizes, reduce the cost of recruitment, and produce more robust results that are clinically relevant. While small sample sizes are common in many studies, they are not appropriate where the patient population is known to be heterogenous [48].

## 5. Recommendations and Future Directions

The eligibility criteria for PLD should be revised to allow for the recruitment of patients with variable diagnostic journeys, facilitate meaningful subgroup analysis, and meet or exceed the average 65% sample yield found in other disease research [10]. These goals can be accomplished by eliminating exclusionary criteria that unnecessarily reduce the sample size without a strong scientific rationale. Specifically, removing the exclusion criteria of patients with prior misdiagnoses of CFS/FMS or psychiatric conditions, as well as the exclusion criteria pertaining to coinfections, would increase the sample yield from 10% to 64% in our model. This approach assumes that screening considers all patients clinically diagnosed with Lyme disease and abandons the restrictive and inappropriate use of the CDC surveillance case definition requirement, while retaining the Rash/WB+ eligibility criteria. This will increase the generalizability of the sample population in these trials to the clinical population.

To further minimize sample attrition, researchers should include a subgroup of patients who do not meet the Rash/WB+ requirement but have been clinically diagnosed with Lyme disease. Clinical diagnosis encompasses other evidence of disease based on patient history, symptom onset, characteristic symptoms and severity, other lab tests, clinical responsiveness to prior treatment, and overall clinical judgment. Including this subgroup would substantially enhance our understanding of PLD, revealing the full spectrum of manifestations and outcomes in patients. It would also illuminate the limitations of relying solely on Rash/WB+ in eligibility criteria. Sample yield can be further improved by replacing functional impairment criteria with symptom-severity measures, which are commonly used in both diagnosis and outcome measures.

To advance research on PLD and ensure that studies address the needs of diverse patient populations, future trials should clearly report eligibility criteria, the scientific rationale behind these criteria, and their impact on sample yield. Researchers should also use RWD to assess recruitment feasibility based on the proposed eligibility criteria to improve trial efficiency and reduce the time and cost to recruit. This will help future researchers optimize trial design and assess feasibility and generalizability.

## 6. Strengths and Limitations

Sources of patient medical information all have strengths and weaknesses. Community-based patient registries such as MyLymeData collect data for the purpose of building a disease-specific knowledge base. Registry data are used in observational studies and cannot demonstrate cause and effect. Our sample population, drawn from the MyLymeData patient registry, contains self-reported patient data where the diagnosis is not independently confirmed by a clinician. This is also true of many government surveys such as BRFSS, NHIS, and other patient registries [9]. However, this study feature may be considered a strength as patients with chronic illnesses have been shown to be a reliable source of information about their conditions when compared to chart review-based studies [49]. Patient registries customarily have fewer eligibility restrictions compared to other data sources, which may result in samples that are more representative of patients seen in clinical practice.

In addition, many Lyme patients (50%) are treated by clinicians whose services are not covered by insurance and who would not be represented in insurance or EHR databases [23]. Although chart review studies can be conducted using EHRs, they are costly, time-consuming, and data from EHRs for an enrollment eligibility study often cannot be compiled because they are not collected in the first place. One study found that of pre-identified elements needed for determining trial eligibility, only 48% could be captured using a standard paper chart review and just 27% of the data elements existed using an EHR data warehouse [11]. In addition to enrollment criteria, EHRs and insurance claims lack patient-reported outcome information such as symptom severity, treatment outcomes, diagnostic history, and quality-of-life indicators [6,23].

In order to be included in the registry, patients require access to an electronic device and an internet connection. Patients are also self-selected; however, this is also true of RCTs and clinical cohort studies in the sense that patients volunteer to participate. Patients who are the sickest, or may have been sick longer, may be more likely to join a patient registry or an RCT as they seek treatment options and information [23]. This study includes over 4000 patients from the MyLymeData patient registry and is significantly larger than other Lyme disease studies, allowing for a robust ALD vs. PLD subgroup analysis [23].

Our survey reflects the point in time when the participant took the survey. Because this is not a longitudinal study, at the time patients take the survey, they identify their stage of disease determined by whether they had been sick for less than six months (ALD) or remained ill for six months or more following antibiotic treatment (PLD), as described in Section 2.2. Therefore, a direct comparison of symptoms between ALD and PLD groups at a specific follow-up time is not possible in this survey.

## 7. Conclusions

Our study highlights the urgent need to improve the external validity of clinical trials in PLD research. The extensive use of eligibility criteria currently excludes approximately 90% of PLD patients from participating in research, greatly reducing the generalizability of findings to real-world clinical populations. Our RWD sample demonstrates that researchers could substantially increase the sample yield from 10% to 64% by loosening restrictions on coinfections and misdiagnoses of chronic fatigue syndrome, fibromyalgia syndrome, and psychiatric conditions.

We also found substantial differences in sample attrition between patients with PLD and those with ALD, further emphasizing the importance of carefully tailoring eligibility criteria for each patient population.

To address these challenges, we propose a more thoughtful approach to generating clinical evidence that balances internal and external validity. Researchers should be parsimonious when selecting eligibility criteria, using them only when there is a strong scientific rationale. Utilizing RWD can help quantify the impact of eligibility criteria on sample generalizability, reduce the time and cost to recruit, and create more inclusive trial designs.

The difference between research samples and the wider PLD patient population raises concerns about the relevance of current research outcomes to clinical practice. It also reflects the divide between academic researchers and community clinicians, which impedes collaborative efforts. Ultimately, our approach aims to bridge the gap between academic research and community clinician practice, ensuring that PLD research has a greater impact on clinical care and improves outcomes for the wider patient population.

## Figures and Tables

**Table 1:** Demographic characteristics.

	PLD ^l^ n (%)	ALD ^2^ n (%)	Diff	*p*-Value ^3^
Sample size	3589	594		
Mean age +/− SD (years) ^a^	51.2 (14.8)	51.2 (16.1)		NS
Sex ^b^ Female	2871 (81%)	448 (76%)	5%	(0.0053)
Male	678 (19%)	142 (24%)	−5%	
Family income ^c^				NS
<$75,000	1286 (51%)	205 (48%)	3%	
≥$75,000	1237 (49%)	220 (52%)	−3%	
US region ^d^				
East	1205 (34%)	268 (45%)	−11%	
Midwest	547 (15%)	112 (19%)	−4%	
South	1067 (30%)	151 (26%)	4%	
West	725 (21%)	58 (10%)	11%	

^1^ Persistent Lyme disease, ^2^ acute Lyme disease, ^3^ alpha = 0.05, Fisher’s Exact Test. Excludes: ^a^ missing (PLD 36, ALD 3), ^b^ missing (PLD 45, ALD 5) and Other (PLD 4, ALD 1), ^c^ missing (PLD 672, ALD 112) and “prefer not to answer” (PLD 394, ALD 67), and ^d^ missing (PLD 45, ALD 5). NS, not significant.

**Table 2:** Prevalence of commonly used eligibility criteria.

	PLD ^l^ n (%)	ALD ^2^ n (%)	Diff	*p*-Value ^3^
Symptoms (≥1 moderate-very severe) ^a^	3224 (98)	488 (93)	5%	(<0.0001)
Rash/WB+ ^b^	2103 (67)	407 (73)	−6%	(<0.0023)
Rash ^c^	1321 (45)	294 (54)	−9%	(<0.0001)
WB+ ^d^	1217 (36)	193 (36)	0	NS
Misdiagnosis ^e^	2516 (74)	237 (43)	31%	(<0.0001)
CFS alone **^4^**	1104 (31)	28 (5)	26%	(<0.0001)
FMS alone **^5^**	1100 (31)	35 (6)	25%	(<0.0001)
Psych alone **^6^**	1361 (38)	65 (11)	27%	(<0.0001)
CFS or FMS	1449 (40)	48 (8)	32%	(<0.0001)
CFS, FMS, or Psych	1892 (53)	94 (16)	37%	(<0.0001)
Coinfections ≥ 1 ^f^	2125 (76)	125 (34)	42%	(<0.0001)
Quality of life				
Activity limited days ≥ 1 ^g^	2687 (93)	403 (90)	3%	(<0.0057)
Bed days ≥ 8 ^h^	1048 (36)	120 (27)	9%	(<0.0001)
SRHS fair/poor ^i^	1884 (74)	138 (36)	38%	(<0.0001)
Disabled ^j^	836 (28)	29 (6)	22%	(<0.0001)
Lyme diagnosis < 1 month ^k^	318 (9)	221 (40)	−31%	(<0.0001)

^l^ Persistent Lyme disease, ^2^ acute Lyme disease, ^3^ all measurable differences are statistically significant (alpha = 0.05, Fisher’s Exact Test), ^4^ chronic fatigue syndrome (CFS), ^5^ fibromyalgia syndrome (FMS), ^6^ psychiatric conditions (Psych). Excludes: ^a^ patients who did not select at least one symptom (PLD 314, ALD 71), ^b^ missing (PLD 442, ALD 36), ^c^ missing (PLD 1, ALD 3) and Don’t Know (DKN) (PLD 671, ALD 51), ^d^ missing (PLD 0, ALD 1) and DKN (PLD 212, ALD 63), ^e^ missing (PLD 1, ALD 1) and DKN (PLD 179, ALD 42), ^f^ missing (PLD 50, ALD 5) and DKN (PLD 725 ALD 218), ^g^ missing (PLD 715, ALD 146), ^h^ missing (PLD 679, ALD 144), ^i^ self-reported health status (SRHS), reduced sample size because separate survey (PLD 1041, ALD 214), ^j^ work status missing (PLD 631, ALD 131), and ^k^ missing (PLD 59, ALD 8). NS, not significant betwenn PLD and ALD groups.

## Data Availability

Data used in the preparation of this article were obtained from the LymeDisease.org patient registry, MyLymeData, Phase 2, 21 August 2022. Restrictions apply to the availability of these data. Data were obtained from LymeDisease.org and are available from the corresponding author subject to the permission of LymeDisease.org, which acts as a data steward on behalf of patients in the MyLymeData patient registry.

## Supplementary Materials

Data supporting calculations in this report are included as supplementary materials available at https://figshare.com/articles/dataset/Supplemental_Materials_12_03_2024_Final_12_3_24_xlsx/27981734?file=51031046 (accessed on 14 October 2024).

## Abbreviations

**AHRQ**	Agency for Healthcare Research and Quality
**ALD**	acute Lyme disease
**BRFSS**	Behavioral Risk Factor Surveillance System
**CDC**	Centers for Disease Control and Prevention
**CFS**	chronic fatigue syndrome
**EHR**	electronic health records
**EM rash**	erythema migrans rash
**FDA**	U. S. Food and Drug Administration
**FMS**	fibromyalgia syndrome
**IRB**	Institutional Review Board
**MCID**	minimal clinically important difference
**NHIS**	National Health Interview Survey
**NIH**	National Institutes of Health
**PCORI**	Patient-Centered Outcomes Research Institute
**PLD**	persistent Lyme disease
**PRO**	patient-reported outcome
**RCT**	randomized controlled trial
**RWD**	real-world data
**RWE**	real-world evidence
**SRHS**	self-reported health status
**WB+**	Western blot positive test result
